# Complete Genome Sequence of Escherichia coli Myophage Mangalitsa

**DOI:** 10.1128/MRA.01045-19

**Published:** 2019-09-19

**Authors:** Cameron L. Atkison, Justin Boeckman, Heather Newkirk, Mei Liu, Jason J. Gill, Jesse Cahill, Jolene Ramsey

**Affiliations:** a Center for Phage Technology, Texas A&M University, College Station, Texas, USA; b Department of Animal Science, Texas A&M University, College Station, Texas, USA; Queens College

## Abstract

Enteropathogenic Escherichia coli is a prevalent Gram-negative bacterium that can lead to fatal complications from infection in humans. Here, we present the isolation and complete annotation of the 52,329-bp genome of enteropathogenic E. coli ATCC 23545 myophage Mangalitsa. Predicted terminal repeats and temperature sensitivity for plaque formation place Mangalitsa with similar unclassified myoviruses.

## ANNOUNCEMENT

Enteropathogenic Escherichia coli (EPEC) strains are Gram-negative human pathogens that are prevalent in both communal and clinical settings (1). They have been identified as one of the leading causes of persistent diarrhea, which is the second largest contributor to childhood mortality, accounting for 1.3 million deaths per year (2, 3). Here, we present the complete genome sequence of enteropathogenic E. coli myophage Mangalitsa.

Bacteriophage Mangalitsa was isolated from a chloroform-sterilized and enriched swine fecal sample collected in College Station, TX, based on its ability to grow on the enteropathogenic E. coli strain ATCC 23545. While the host was typically grown aerobically at 37°C in Luria broth (BD) and standard soft agar overlay methods were used (4), Mangalitsa only produced plaques at 30°C or 22°C. Phage genomic DNA was isolated using the shotgun library prep modification of the Promega Wizard DNA clean-up system (5). A genomic library prepared with the TruSeq Nano low-throughput kit was sequenced by an Illumina MiSeq platform with paired-end 250-bp reads. A total of 480,501 reads were in the phage index. Quality control was performed with FastQC (http://www.bioinformatics.babraham.ac.uk/projects/fastqc/). Sequence reads were then trimmed using the FASTX-Toolkit v0.0.14 (http://hannonlab.cshl.edu/fastx_toolkit/). The genome was assembled with 1,333-fold coverage using SPAdes v3.5.0 and closed by PCR (forward primer, 5′-AGTGCACGGTATTCTTCGTTAG-3′; reverse primer, 5′-CTAACGCATCGAATCTCTTCTCA-3′) and Sanger sequencing (6). Structural annotations were made with GLIMMER v3.0 and MetaGeneAnnotator v1.0, and ARAGORN v2.36 did not reveal any tRNAs (7–9). Protein-coding gene function was predicted with InterProScan v5.33-72 and BLAST v2.2.31 (10, 11). The BLAST analysis queried the NCBI nonredundant and UniProtKB Swiss-Prot and TrEMBL databases with a 0.001 maximum expectation value cutoff (12). Rho-independent termination sites were analyzed using TransTermHP v2.09 (13). Whole-genome similarity was calculated by the progressiveMauve v2.4.0 algorithm (14). The annotation tools used are in the Galaxy and Web Apollo instances hosted by the Center for Phage Technology (https://cpt.tamu.edu/galaxy-pub). The morphology of the phage sample was determined by negative staining with 2% uranyl acetate and visualized with transmission electron microscopy at the Texas A&M University Microscopy and Imaging Center (15).

Mangalitsa is a myophage with a 52,329-bp genome consisting of 44.01% GC content, 1 terminator, 0 tRNAs, and 82 predicted protein-coding genes. Of the protein-coding genes, 31 have a predicted function, while 51 are hypothetical proteins, totaling an overall 89.66% coding density. Additionally, Mangalitsa has 3,234-bp terminal repeats detected by PhageTerm (16). The terminal repeat pattern is consistent with its most closely related phages, which include enterobacterial phage phiEcoM-GJ1 (79.61% nucleotide similarity; GenBank accession no. EF460875), Pectobacterium phage PP101 (47.01% nucleotide similarity; KY087898), *Pectobacterium* phage PM1 (46.24% nucleotide similarity; KF534715), and Erwinia phage vB_EamM-Y2 (37.28% nucleotide similarity; HQ728264) (17–19). As such, the Mangalitsa genome was reopened with the RNA polymerase gene as the first feature following the terminal repeat to follow the convention for this group of phages. Interestingly, temperature sensitivity was also reported for the related *Escherichia* myophage ST32 (MF044458) (20).

### Data availability.

The genome sequence and associated data for phage Mangalitsa were deposited under GenBank accession no. MN045229, BioProject no. PRJNA222858, SRA no. SRR8869233, and BioSample no. SAMN11360419.
